# The paradoxical role of MDSCs in inflammatory bowel diseases: From bench to bedside

**DOI:** 10.3389/fimmu.2022.1021634

**Published:** 2022-09-15

**Authors:** Fan Zhao, Wenbin Gong, Jiaojiao Song, Zhe Shen, Dawei Cui

**Affiliations:** ^1^ Department of General Surgery, The First Affiliated Hospital, Zhejiang University School of Medicine, Hangzhou, China; ^2^ Department of General Surgery, The First Affiliated Hospital of Xi’an Jiaotong University, Xi’an, China; ^3^ Department of Gastroenterology, The First Affiliated Hospital, Zhejiang University School of Medicine, Hangzhou, China; ^4^ Department of Blood Transfusion, The First Affiliated Hospital, Zhejiang University School of Medicine, Hangzhou, China

**Keywords:** myeloid-derived suppressor cells, immune regulation, immunosuppressive function, intestinal inflammation, inflammatory bowel disease

## Abstract

Myeloid-derived suppressor cells (MDSCs) are a group of bone marrow derived heterogeneous cells, which is known for their immunosuppressive functions especially in tumors. Recently, MDSCs have receiving increasing attention in pathological conditions like infection, inflammation and autoimmune diseases. Inflammatory bowel diseases (IBD) are a series of immune-dysfunctional autoimmune diseases characterized by relapsing intestinal inflammation. The role of MDSCs in IBD remains controversial. Although most studies *in vitro* demonstrated its anti-inflammatory effects by inhibiting the proliferation and function of T cells, it was reported that MDSCs failed to relieve inflammation but even promoted inflammatory responses in experimental IBD. Here we summarize recent insights into the role of MDSCs in the development of IBD and the potential of MDSCs-targeted therapy.

## Introduction

Myeloid-derived suppressor cells (MDSCs) are a general term for the group of heterogeneous cells derived from bone marrow, which are generally considered to be immunosuppressive especially in a tumor-related context by inhibiting T cells and regulating immune responses (1). The term was first introduced by Gabrilovich in 2007 (2). Before 2007, these cells were described in various studies and named as natural suppressor cells or immature myeloid cells (IMCs) (3–7). Normally, IMCs act as the precursors of dendritic cells, macrophages and granulocytes to regulate immune responses. Few IMCs can be detected in the peripheral blood of healthy individuals. However, under pathological conditions such as tumors, infection or inflammation, the maturation of IMCs is interrupted. IMCs remain at different stages of differentiation, namely MDSCs (8–10). Commonly, MDSCs are divided into two groups according to their phenotypic features: granulocyte-like MDSCs (G-MDSC or PMN-MDSC) and monocyte-like MDSCs (M-MDSCs). For mice, MDSCs are considered to be CD11b^+^Gr-1^+^ and divided into G-MDSCs and M-MDSCs according to their binding specificity to different epitopes (Ly6G and Ly6C) of Gr-1, a myeloid differentiation antigen. Murine G-MDSCs are usually considered to be CD11b^+^Ly6G^+^Ly6C^low^ while M-MDSCs are CD11b^+^Ly6G^-^Ly6C^high^. Given the absence of Gr-1 genes in human, human G-MDSCs are considered as CD11b^+^CD14^-^CD15^+^ while M-MDSCs are HLA-DR^-/low^CD11b^+^CD14^+^CD15^-^ (7–9, 11). There also exists a subpopulation of human MDSCs named early-stage MDSCs (eMDSCs), immature MDSCs or promyelocytic MDSCs. eMDSCs comprise more immature progenitors and are considered to be HLA-DR^-^CD33^+^CD11b^+^Lin-1^-^ in human (8). G-MDSCs and M-MDSCs are more differentiated than eMDSCs, but lack efficient markers to be differed from the mature fraction of monocytes and granulocytes.

Under pathological conditions, MDSCs proliferate greatly and are recruited to the lesion area to elicit their functional roles (10, 12–15). The expansion of MDSCs is mainly regulated by the JAK/STAT3 pathway (16–18). Cytokines inducing the expansion of MDSCs include prostaglandin E (PGE), cyclooxygenase-2 (COX2), prostaglandin-like stem cell factor (SCF), macrophage colony-stimulating factor (M-CSF), IL-6, granulomonocyte colony-stimulating factor (GM-CSF) and vascular endothelial cell growth factor (VEGF) (19–23). In addition to the expansion of MDSCs, the activation of MDSCs is also important for exerting their immune functions. Cytokines such as IFN-γ, and TGF-β are produced by activated T cells, impaired bacterial or tumor cells, and activate different signaling pathways in MDSCs (24–28). Blockade of these cytokines can remove MDSCs-mediated immunosuppression by inhibiting related signals such as STAT6, STAT1 and NF-κB (29–32).

Commonly, MDSCs are considered to be immunosuppressive and inhibit adaptive immune responses by inhibiting T cells or inducing Treg cells (21, 33–35). MDSCs can also inhibit innate immune responses by inhibiting the cytotoxicity of natural killer cells (NK cells), polarizing macrophages towards the anti-inflammatory phenotype *via* TGF-β, IL-10 and so on (36–38). However, the role of MDSCs is not always immunosuppressive. MDSCs were observed to activate NK cells by expressing RAE-1 (Retinoic acid early transcript 1) ligands for the NK cell group 2 member D receptor, which in turn dampened MDSCs (39). The contrast results may result from different phenotypes of MDSCs. Therefore, identifying specific molecules expressed by MDSCs to elicit immunosuppressive functions can be one of the future directions.

Inflammatory bowel diseases (IBD), consisted of Crohn’s disease (CD) and ulcerative colitis (UC), are an autoimmune disease characterized by chronic, transmural and relapsing gastrointestinal inflammation, and associated with a high risk of developing colorectal cancer (40–42). IBD can be divided into active and remission stages according to the disease activity. The etiology and detailed mechanism of the imbalance between anti-inflammatory and pro-inflammatory states remain to be elusive in IBD, making it still incurable today. Extensive infiltration of immune cells in intestines has been observed in IBD, reflecting their crucial roles in the disease development (43–45). Therefore, as an important immune cell, the potential role of MDSCs in IBD has gradually attracted attention from researchers, and controversial results were observed. In the review, we will demonstrate the existing studies of MDSCs in IBD reporting either its pro-inflammatory roles or anti-inflammatory roles, along with the application of MDSCs targeted therapies in IBD.

## The dual role of MDSCs in the development of IBD

### Human MDSCs increased in IBD and exhibited paradoxical effects on T cells *in vitro*

Human MDSCs denoted as CD14^+^HLA-DR^-/low^ cells were first observed to accumulate in the peripheral blood of patients with active Crohn’s disease (CD) and ulcerative colitis (UC) in 2008 (46). This study enrolled 18 active UC, 21 active CD and 12 healthy controls. Compared with MDSCs from healthy controls, MDSCs from IBD patients exhibited more powerful immunosuppressive function by expressing arginine-1 (Arg-1) and inhibiting the IFN-γ release of anti-CD3/CD28-stimulated autologous peripheral blood mononuclear cells *in vitro*. The above findings were corroborated by another study in 2015, in which 33 CD patients including 16 active and 17 inactive ones, 36 UC patients including 21 active and 15 inactive ones, and 36 healthy controls were enrolled (47). In the study, CD14^+^HLA-DR^-/low^ cells were identified as human M-MDSCs, while CD33^+^CD11b^+^HLA-DR^-^ cells were identified as G-MDSCs. Consistent with the previous study, more M-MDSCs, namely CD14^+^HLA-DR^-/low^ cells, were observed in the peripheral blood of IBD patients. Besides, its frequency was positively correlated with the disease activity. However, as for G-MDSCs, namely CD33^+^CD11b^+^HLA-DR^-^ cells, researchers demonstrated that no significant difference was found between healthy controls and CD or UC patients. Although the study reported opposite results concerning human G-MDSCs and M-MDSCs in IBD, it should be noticed that the identification of G-MDSCs was not standard enough. Following the definition of M-MDSCs in this study, another study conducted in 2021 confirmed the significant increase of CD14^+^HLA-DR^-/low^ MDSCs in UC by examining 36 UC patients and 34 healthy controls (48). On the other hand, another study conducted in 2017 focused on CD33^+^CD15^+^CD14^–^HLA-DR^–/low^ MDSCs in IBD patients (49). The phenotype of MDSCs in the study is similar to G-MDSCs studied in the previous study (47). The study enrolled 25 UC patients including 12 active and 13 inactive ones, 22 CD patients including 11 active and 11 inactive ones, as well as 19 healthy controls. Significant differences in the frequency and number of CD33^+^CD15^+^CD14^–^HLA-DR^–/low^ MDSCs were observed between healthy controls and active CD or UC patients. No significant difference was observed between healthy controls and inactive CD or UC. Moreover, CD33^+^CD15^+^CD14^–^HLA-DR^–/low^ MDSCs purified from the peripheral blood of IBD patients promoted the proliferation of autologous sorted CD4^+^CD25^–^ T cells *in vitro*. The findings raised an intriguing question about the function of MDSCs in IBD. Overall, the above studies demonstrated the accumulation of MDSCs in human IBD, and revealed that CD14^+^HLA-DR^-/low^ MDSCs from the peripheral blood of IBD patients inhibited anti-CD3/CD28–stimulated autologous mononuclear cells while CD33^+^CD15^+^CD14^–^HLA-DR^–/low^ MDSCs promoted the proliferation of autologous T cells. However, there remained a paucity in the field of human MDSCs in IBD. The identification of MDSCs including G-MDSCs and M-MDSCs varied in the above studies and the population sizes in the studies were relatively small. More studies are required to explore different functional roles of the subsets of MDSCs in IBD patients based on a more acute classification.

### Murine MDSCs accumulated in experimental IBD but failed to inhibit the pro-inflammatory effects of T effector cells

Despite of few studies of MDSCs in IBD patients, a number of studies employed murine colitis models to explore the functional roles of MDSCs. Dextran sulfate sodium (DSS)-induced, 4,6-trinitrobenzene sulfonic acid (TNBS)-induced, T cell transfer-induced or genetic defects-induced spontaneous murine colitis models were established to mimic the pathology of IBD (50). Murine MDSCs were identified as CD11b^+^Gr-1^+^ cells in most studies. Besides, G-MDSCs were identified to be CD11b^+^Ly6G^+^ and M-MDSCs were to be CD11b^+^Ly6C^+^. Haile *et al.* first identified the immunosuppressive role of MDSCs by employing DSS-induced and T cell transfer-induced murine colitis models in VILLIN-hemagglutinin (HA) transgenic mice of BALB/c background (46). The researchers showed that repetitive transfer of clone 4/T-cell receptor (CL4-TCR) transgenic CD8^+^ T cells into VILLIN-HA mice led to a significant increase in MDSCs frequency in spleens and intestines compared with a single transfer of T cells. Consistently, repetitive T cell transfer decreased the severity of inflammation. However, MDSCs frequency did not significantly increase in spleens or mesenteric lymph nodes in DSS-induced VILLIN-HA transgenic mice. The increase of GM-CSF and IFN-γ in intestines was only observed in T cell transfer-induced rather than DSS-induced VILLIN-HA transgenic mice, implying that GM-CSF and IFN-γ were involved in MDSC generation. Consistently, several studies confirmed the increase of MDSCs in the peripheral blood, bone marrow and spleens by establishing chronic colitis in C.B-17 SCID mice after adoptive transfer of CD4^+^CD45RB^high^ T cells, or in BALB/c mice after DSS administration (47, 51). Besides, it was reported that the number of MDSCs significantly increased especially at the recovery phase of DSS-induced chronic colitis, thus contributing to the alleviation of inflammation (52, 53). As for the acute colitis induced by DSS or TNBS, the proportion of CD11b^+^Gr-1^+^ MDSCs and its subsets (CD11b^+^Ly6C^+^ M- and CD11b^+^Ly6G^+^ G-MDSCs) also increased in spleens and lamina propria and positively correlated with intestinal inflammatory scores (54).

To explore the interaction between MDSCs and T cells, Kurmaeva *et al.* isolated CD11b^+^Ly6G^-^Ly6C^high^ MDSCs, namely M-MDSCs from recombination-activating gene 1 deficient (RAG-1^-/-^) mice with chronic colitis induced by T cell transfer, and added the group of M-MDSCs to activated CD4^+^ T cells *in vitro* (55). As expected, M-MDSCs suppressed T cell proliferation at a 1:1 ratio, while other subsets of CD11b^+^Gr-1^+^ MDSCs did not. Besides, the suppression was reversed after administrating an inducible nitric oxide synthase (iNOS)-specific inhibitor or immunoneutralization of IFN-γ, other than an arginase-1 inhibitor or excess of L-arginine. Further experiment revealed that the inhibition of T cells by M-MDSCs was mediated by cell contact, nitric oxide (NO), partially IFN-γ and prostaglandins. Furthermore, the co-culture of MDSCs and CD4^+^ T cells enhanced the generation of Th17 and foxp3^+^ T cells. However, the co-culture of MDSCs and Th1/Th17 CD4^+^ T effector cells isolated from inflamed colon lamina propria had no effect on the levels of IFN-γ, IL-2, IL-6, IL-17 and GM-CSF although the proliferation of T effector cells was inhibited. Taken together, the researchers identified the immunosuppressive function of MDSCs to restrain Th1 responses and trigger the polarization of activated T cells towards Th17 or an inducible Treg phenotype, which was not sufficient to prevent the development of colitis. All studies mentioned above mostly drew conclusions based on *in vitro* and gene-expression data, so that their conclusions are limited. Overall, the activation of MDSCs may be aimed at achieving homeostasis by suppressing the over activation of T cells but not capable of switching the immune environment in chronic inflammation into an anti-inflammatory state.

Although most studies proposed the immunosuppressive effect of MDSCs, several studies illustrated their pro-inflammatory roles under the inflammatory conditions of murine colitis. Varol *et al.* reported that adoptively transferred bone marrow-derived CD11b^high^Ly6C^high^ MDSCs were transformed into pro-inflammatory CX3CR1^+^CD11b^+^ dendritic cells in mice with systemic depletion of intestinal dendritic cells (56). Moreover, these cells promoted intestinal inflammation after DSS administration. Rivollier *et al.* isolated CD115^+^CD11b^high^Ly6C^high^ cells from the bone marrow of congenic (CD45.1^+^) mice and adoptively transferred them into recipient steady-state mice and chronic colitis mice (57). The results showed that these cells were selectively differentiated into anti-inflammatory F4/80^high^CX3CR1^high^CD11c^+^CD11b^+^CD103^-^ macrophages in noninflamed colons and pro-inflammatory CD103^-^CX3CR^int^CD11b^+^ dendritic cells in inflamed colons respectively. Similarly, Ostanin *et al.* found that CD11b^+^Ly6C^int^Gr-1^+^ cells isolated from colons of chronic colitis mice expressed enhanced levels of MHC-II and CD86, acting as antigen-presenting cells to induce T cell activation *in vitro* (58). The above studies claimed that MDSCs exerted a pro-inflammatory effect by differentiating into pro-inflammatory dendritic cell-like cells under the inflammatory environment. As mentioned earlier, Kontaki *et al.* declared in 2017 that CD33^+^CD15^+^CD14^–^HLA-DR^–/low^ MDSCs isolated from IBD patients enhanced autologous T cell proliferation (49). They also established a TNBS-induced acute colitis model in Balb/c mice. CD11b^+^Gr-1^+^Ly6G^+^ MDSCs, namely G-MDSCs, increased significantly in the acute phase of TNBS-induce colitis while CD11b^+^Gr-1^+^Ly6C^+^ MDSCs, namely M-MDSCs, did not. MDSCs were isolated from bone marrows and spleens of colitis mice to detect the level of C/EBPβ, a master regulator of MDSCs’ suppressive function (59). Subsequently the absence of C/EBPβ suggested the loss of immunosuppressive function of MDSCs and implied that complex inflammatory environment *in vivo* altered the suppressive capacity of MDSCs. In addition, Tachibana *et al.* established a IL10^-/-^IL17A^-/-^ spontaneous murine colitis model, eliciting a stronger inflammatory storm than the IL10^-/-^ spontaneous murine colitis model (60). Noteworthy is that, the number of MDSCs including G-MDSCs and M-MDSCs also significantly increased in the bone marrows, spleens and peripheral blood of IL10^-/-^IL17A^-/-^ mice. Both G-MDSCs and M-MDSCs expressed more iNOS and less Arg-1, releasing NO into the circulation to exacerbate colitis, probably relying on NO-induced disruption of the gut microbiota. Cell experiments revealed that G-MDSCs from IL10^-/-^IL17A^-/-^ mice strongly inhibited the proliferation of CD4^+^ T cells but weakly inhibited the proliferation of CD8^+^ T cells. Furthermore, IFN-γ derived from CD8^+^ T cells enhanced the expression of iNOS in G-MDSCs to exacerbate inflammation in IL10^-/-^IL17A^-/-^ mice.

Generally, these studies demonstrated the dual role of MDSCs in colitis and were briefly summarized in **Table 1**. Although most studies identified the immunosuppressive role of MDSCs in experimental IBD, the functional plasticity of heterogeneous populations was also observed. The disparity in the conclusions could be attributed to different disease stages, mice background, modeling time, drug brand and dosage used for modeling, and the difficulty in differing MDSCs from neutrophils phenotypically. Currently, most studies of MDSCs in IBD focused on its interaction with adaptive immune cells like T cells, Th1 cells, Th17 cells and Treg cells. A few studies demonstrated the relationship between MDSCs and innate immune cells like dendritic cells and macrophages. For adaptive immune cells, MDSCs inhibited the proliferation of CD4^+^ T cells at a 1:1 ratio by cell contact, NO, IFN-γ and prostaglandins and triggered the polarization of T cells towards Th17 or an inducible Treg phenotype (55). In turn, Treg cells inhibited the conversion of MDSCs into G-MDSCs depending on the expression of Tgfbr2 genes in MDSCs and secreted TGF-β to promote the expansion of MDSCs (61). MDSCs could also elicit its functional roles by secreting exosomes. Therefore, it was reported that the frequency of Th1 cells decreased after the administration of G-MDSCs-derived exosomes in experimental IBD (62). For innate immune cells, MDSCs inhibited the migration of CD11b^+^Ly6C^+^ macrophages towards colon lamina propria in order to restrict inflammation in IBD (52). However, MDSCs from inflamed colon lamina propria of IBD failed to inhibit the inflammatory effects of T cells (55). Furthermore, they could be differentiated into dendritic cell-like cells, acting as antigen-presenting cells to induce T cell activation (56–58). The relationship between MDSCs and NK cells has not been studied in IBD yet whereas other studies demonstrated the crosstalk between MDSCs and NK cells in tumors (36–39).

**Table 1:** Phenotypes and Roles of MDSCs in IBD.

Colitis Model	Colitis Stage	Cell phenotype	General Function	Published Year	Reference
T cell transfer/DSS induced	chronic	Human MDSCs: CD14^+^CD19^-^HLA-DR^-/low^ Murine MDSCs: CD11b^+^Gr-1^+^	Anti-inflammatory	2008	(46)
DSS induced	chronic	Human M-MDSCs: CD14^+^ HLA-DR^-/low^ Human G-MDSCs: CD11b^+^CD33^+^HLA-DR^-^ Murine MDSCs: CD11b^+^Gr-1^+^	Anti-inflammatory	2015	(47)
T cell transfer induced	chronic	Murine MDSCs: CD31^+^CD11b^+^Gr-1^+^	Anti-inflammatory	2008	(51)
DSS induced	acute	Murine MDSCs: CD11b^+^Gr-1^+^	Anti-inflammatory	2011	(52)
DSS induced	chronic	Murine MDSCs: CD11b^+^Gr-1^+^	Anti-inflammatory	2011	(53)
TNBS induced	chronic	Murine MDSCs: CD11b^+^Gr-1^+^	Anti-inflammatory	2013	(54)
T cell transfer induced	chronic	Murine G-MDSCs: CD11b^+^Gr-1^+^Ly6G^+^	Anti-inflammatory	2014	(55)
DSS induced	acute	Murine M-MDSCs: CD11b^+^Ly6C^+^	Pro-inflammatory	2009	(56)
T cell transfer induced	chronic	Murine M-MDSCs: CD115^+^CD11b^+^Ly6C^+^	Pro-inflammatory	2012	(57)
T cell transfer induced	chronic	Murine G-MDSCs: CD11b^+^Ly6G^+^Ly6C^int^	Pro-inflammatory	2012	(58)
TNBS induced	acute	Human MDSCs: CD14^-^CD15^+^CD33^+^HLA-DR^-/low^ Murine MDSCs: CD11b^+^Gr-1^+^	Pro-inflammatory	2017	(49)
IL10^-/-^IL17A^-/-^ spontaneous	acute	Murine MDSCs: CD11b^+^Gr-1^+^	Pro-inflammatory	2020	(60)

## MDSCs-targeted therapies alleviated chronic inflammation in experimental IBD

### The depletion of MDSCs aggravated inflammation in murine colitis

Considering the immunosuppressive function of MDSCs, murine colitis models were employed to detect the effects of different MDSCs-targeted methods on IBD. In general, there were three methods to target MDSCs, namely, the use of antibodies to deplete MDSCs, the use of molecules to regulate the expansion or differentiation of MDSCs, and the transfer of MDSCs or vesicles secreted by MDSCs into colitis models. The corresponding studies are summarized in **Table 2**. For studies using antibodies to deplete MDSCs, it should be noted that other immune cells expressing the antibody-specific markers were also removed. In 2007, Hoffmann *et al.* depleted MDSCs in colitis mice by intraperitoneal injection of anti-Gr-1 mAb and depleted MDSCs in colitis rats by rabbit polyclonal antiserum against polymorphonuclear neutrophils prior to colitis induction (77). The results showed that colitis was aggravated after the depletion of MDSCs, exhibiting a more rapid weight loss, increased mortality and increased bacterial translocation into cardiac blood, spleen, and mesenteric lymph nodes. Moreover, Nemoto *et al.* confirmed the detrimental role of MDSCs depletion by long-term administration of anti-Gr-1 mAb to colitic mice at 3 weeks after establishing colitis, which exacerbated inflammation with a systemic expansion of activated CD4^+^ T cells (51). The aggravation of colitis after MDSCs depletion was further confirmed in chemical-induced colitis by injecting anti-Gr-1 mAb intraperitoneally after colitis induction or performing splenectomy to deplete MDSCs prior to colitis induction (49, 53, 70–72).

**Table 2:** MDSCs-targeted therapies in IBD.

Colitis Model	Colitis Stage	Key molecules	Therapeutic Effect	Published Year	Reference
IL10^-/-^spontaneous	chronic	Oral administration of Resveratrol	Anti-inflammatory	2012	(63)
DSS induced	acute	Intraperitoneal injection of Quercetin	Anti-inflammatory	2020	(64)
DSS induced	acute	Oral administration of Butyrate	Anti-inflammatory	2021	(65)
DSS induced	acute	Deficiency of Protein tyrosine phosphatase 1b as immune mediator	Anti-inflammatory	2013	(66)
DSS induced	acute	Knock-in of Glycoprotein130 receptor	Anti-inflammatory	2016	(67)
DSS induced	acute	Intraperitoneal injection of MSCs expressing IL37b	Anti-inflammatory	2015	(68)
Pdk1^flox/flox^CD4-Cre spontaneous	chronic	Intravenous injection of Treg cells	Anti-inflammatory	2016	(61)
DSS induced	acute	Oral administration of A mTOR kinase inhibitor	Anti-inflammatory	2019	(69)
DSS induced	acute	Intravenous injection of An inhibitor of enhancer of zeste homolog 2	Anti-inflammatory	2019	(70)
IL10^-/-^spontaneous	chronic	Deficiency of IL17A	Pro-inflammatory	2020	(60)
DSS induced	acute	Enema administration of Acetylcholine	Anti-inflammatory	2021	(71)
DSS induced	acute	Intraperitoneal injection of An endothelin-A receptor antagonist	Anti-inflammatory	2021	(72)
DSS induced	acute	Intraperitoneal injection of A cannabinoid receptor 2 inverse agonist	Anti-inflammatory	2022	(73)
DSS induced	acute/chronic	Intraperitoneal injection of Atorvastatin (drug)	Anti-inflammatory	2016	(74)
DSS induced	chronic	Intraperitoneal injection of Sildenafil	Anti-inflammatory	2017	(75)
T cell transfer induced	chronic	Transfer of MDSCs	Anti-inflammatory	2008	(46)
DSS induced	acute/chronic	Transfer of MDSCs	Anti-inflammatory	2011, 2016, 2020, 2013, 2021, 2016	(52, 61, 64, 66, 72, 74)
TNBS induced	chronic	Transfer of MDSCs	Anti-inflammatory	2013	(54, 76)
TNBS induced	acute	Transfer of MDSCs	Pro-inflammatory	2017	(49)
DSS induced	acute	Transfer of G-MDSCs-derived exosome	Anti-inflammatory	2016	(62)
TNBS/DSS induced, T cell transfer induced	acute/chronic	Depletion of MDSCs	Pro-inflammatory	2017, 2008, 2011, 2019, 2021, 2021, 2007	(49, 51, 53, 70–72, 77)

### Bioactive molecules regulated MDSCs to treat murine colitis

A number of studies illustrated the administration of different molecules to treat colitis by regulating MDSCs. Several studies paid attention to natural products. Nagarkatti *et al.* found a significant expansion of MDSCs triggered by resveratrol and identified resveratrol as a potential therapeutic approach for IBD (63). In the study, resveratrol was orally administrated to IL10^-/-^ spontaneous colitis mice every second day from the 18th weeks of chronic colitis. The number of MDSCs in spleens and colon lamina propria increased after resveratrol treatment. The majority of the MDSCs expressed Arg-1 and minimal levels of iNOS and were more potent at inhibiting T cells. As expected, the number of CD4^+^CD69^+^, CD4^+^CXCR3^+^ and CD4^+^CXCR10^+^ T cells decreased and the colon inflammation was dampened. Ma *et al.* illustrated that the injection of quercetin, a natural product ubiquitously existing in leaves, fruits or seeds of plants, enhanced the proliferation and the phosphorylation level of MDSCs to alleviate acute colitis after binding to the estrogen receptor (64). Butyrate, derived from anaerobic fermentation of indigestible starch and fibrin in the intestinal lumen, was orally administrated into a DSS-induced colitis model according to the study conducted by Xiao *et al.* (65). Butyrate assisted MDSCs in alleviating colitis and ultimately reduced the compensatory recruitment of MDSCs to the inflamed colon by inhibiting the upregulation of chemokine CC motif receptor 9 (CCR9).

In addition to natural products, the mediators of immune system were also examined and various inhibitors or agonists were administrated in colitis models. The deficiency of protein tyrosine phosphatase 1b enhanced the expansion of MDSCs by promoting the activity of STAT3 and thus alleviated the inflammation of DSS-induced colitis models (66). Knock-in of glycoprotein130 receptor prevented DSS-treated mice from developing acute colitis by activating STAT3 and promoting the expansion of G-MDSCs dramatically (67). Wang *et al.* transferred IL-37b gene into mesenchumal stromal cells (abbreviated as MSC-IL37b) and injected MSC-IL37b to DSS-induced colitis models (68). The results showed that the proportion of MDSCs in total splenic mononuclear cells as well as that of Tregs in splenic CD4^+^ cells significantly increased in MSC-IL-37b-treated colitis mice. IL-37b produced by MSC-IL-37b indirectly promoted the differentiation of MDSCs and Tregs in spleens. Lee *et al.* illustrated the interaction between Treg cells and MDSCs in IBD in 2016 (61). The study introduced Pdk1^flox/flox^CD4-Cre mice that can spontaneously develop colitis on the account of impaired function of Tregs. MDSCs from the Pdk1^flox/flox^CD4-Cre mice exhibited a weaker immunosuppressive function, including lower expression of iNOS, weaker suppression of CD4^+^ T cell proliferation and less efficient function to induce Treg cells *in vitro*. Adoptive transfer of wild-type Treg cells into Pdk1^flox/flox^CD4-Cre mice repaired the immunosuppressive function of MDSCs and attenuated colitis. Consistently, the total number of MDSCs increased, in which the proportion of G-MDSCs reduced while that of M-MDSCs increased, implying the regulatory function of Tregs in MDSCs expansion and differentiation. Besides, researchers demonstrated that Tregs-mediated inhibition of M-MDSCs conversion into G-MDSCs relied on the expression of Tgfbr2 genes in MDSCs. Moreover, in addition to regulating MDSCs differentiation, Tregs-derived TGF-β played a crucial role in enhancing the immunosuppressive function of MDSCs. Besides, M-MDSCs promoted the expansion of Tregs *in vivo* and *in vitro*. Therefore, a positive feedback loop between Tregs and MDSCs was established during murine colitis. Shi *et al.* revealed that INK128, an inhibitor of mammalian target of rapamycin (mTOR), inhibited the differentiation of MDSCs into mature macrophages and elicited the role of MDSCs to inhibit Th1 response and promote Tregs expansion (69). Besides, INK128 promoted the production of IFN-α which enhanced the immunosuppressive function of MDSCs by elevating the levels of Arg-1 and reactive oxygen species (ROS). Therefore, oral administration of INK128 attenuated DSS-induced colitis by targeting MDSCs. Zhou *et al.* demonstrated that GSK343, a selective inhibitor of enhancer of zeste homolog 2 (EZH2), increased MDSCs in the colonic lamina propria, bone marrows and peripheral blood and alleviated colitis (70). So did GSK126, another EZH2 inhibitor that was evaluated in clinical trials to treat cancer (ClinicalTrials.gov identifier: NCT02082977). *In vitro* experiments revealed that GSK343 promoted the generation of MDSCs from hematopoietic progenitor cells. Tachibana *et al.* illustrated that IL17A, a representative cytokine released by Th17 cells, negatively regulated the accumulation of G-MDSCs which was cooperatively promoted by IL17F and serum amyloid A1/2 (SAA1/2) in an IL-10-defcient background (60). Zheng *et al.* demonstrated that acetylcholine (ACh), a neurotransmitter that decreased in IBD as a result of the impaired enteric nervous system, regulated the differentiation of MDSCs by selectively increasing the frequency of M-MDSCs instead of G-MDSCs both *in vivo* and *in vitro* (71). ACh promoted the survival of M-MDSCs under 5-fluorouracil induced apoptosis and elevated the expression of C-C motif chemokine receptor 2 (CCR2) on M-MDSCs which implied the recruitment of MDSCs from circulation. These results partially account for the increase of M-MDSCs after ACh stimulation. Besides, ACh enhanced the IL-10 expression of M-MDSCs by activating the nAChR/ERK pathway, contributing to the inhibition of T cells. ACh administration in DSS-induced acute colitis models *via* enema significantly increased MDSCs in the colon lamina propria rather than that in the circulation or spleens, suggesting that only local immune environment in colons, instead of systemic immunity, was affected by ACh. Consistently, ACh treatment elicited MDSCs to decrease the generation of Th17 cells in colons and thus ameliorated DSS-induced colitis. The intraperitoneal injection of BQ123, an endothelin-A receptor antagonist, increased G-MDSCs rather than M-MDSCs in healthy mice (72). Meanwhile, BQ123 injection activated the suppressive function of G-MDSCs to alleviate DSS-induced acute colitis *via* IL13/STAT6/ARG1 pathway. Moreover, the intraperitoneal injection of SMM-189, a cannabinoid receptor 2 (CB2) inverse agonist, increased the number of MDSCs, reduced Th17 cells and alleviated acute colitis (73).

Interestingly, there were studies investigating the potential effect of drugs that were widely used in clinical practice. Lei *et al.* demonstrated that atorvastatin promoted the expansion of both human HLA-DR^-/low^CD11b^+^CD33^+^ MDSCs and murine CD11b^+^Arg-1^+^ MDSCs *in vitro* (74). The co-culture of atorvastatin significantly promoted the differentiation of GM-CSF-stimulated murine bone marrow cells towards G-MDSCs rather than M-MDSCs, and decreased the frequency of CD11c^+^MHC-II^+^ dendritic cells. It was reported that the expansion of MDSCs by atorvastatin depended on the mevalonate pathway. Similarly, *in vivo* experiments revealed that the number of G-MDSCs rather than M-MDSCs significantly increased in livers, spleens and peripheral blood of healthy or colitic mice after the intraperitoneal injection of atorvastatin. Besides, *in vitro* T cell proliferation assays revealed the immunosuppressive functions of these MDSCs. However, the suppressive function was inhibited after adding an iNOS inhibitor, illustrating that atorvastatin-derived MDSCs depended on NO to inhibit the proliferation of T cells. In addition, the administration of sildenafil, an inhibitor of phosphodiesterase-5, alleviated DSS-induced colitis and reduced the number of iNOS^+^ G-MDSCs in the colons by blocking their recruitment (75).

### Transfer of MDSCs or MDSCs-derived exosomes was administrated in murine colitis

Attempt to adoptively transfer MDSCs into colitis models was first made in 2008 by Haile *et al.* (46). MDSCs were isolated from murine colitis induced by repetitive T cell transfer, and were next co-transferred with HA-specific CD8^+^ T cells into naive VILLIN-HA mice. The inflammation was significantly ameliorated, indicating an anti-inflammatory effect of MDSCs on colitis induced by antigen-specific T cells. Intraperitoneal injection of MDSCs isolated from spleens of colitis mice at the convalescent phase dampened inflammatory responses in DSS-induced acute colitis models by inhibiting the migration of CD11b^+^ single positive population to colon lamina propria. It was reported that the CD11b^+^ single positive cells were composed of Ly6C^high^Ly6G^low^ macrophages (52). In 2012, Su *et al.* reported that bone marrow derived G-MDSCs isolated from healthy mice were intravenously injected to TNBS-induced mice and attenuated colitis by decreasing the level of IL-6 in serum and the activity of myeloperoxidase (MPO) in colons (76). Besides, the accumulation of injected G-MDSCs was more significant in inflamed colons than in the spleens and livers, revealing their tendency to migrate towards inflammatory sites *in vivo*. Consistently, Zhang *et al.* demonstrated that bone marrow derived MDSCs from healthy mice were injected intravenously after colitis induction by DSS and significantly attenuated colitis (66). Guan *et al.* also confirmed the therapeutic effects of transferred MDSCs either isolated from colitis mice or generated *in vitro* on TNBS-induced colitis, which downregulated the expression of pro-inflammatory cytokines in colons (54). In the study, the intravenous injection of MDSCs was prior to TNBS administration, and the total number of MDSCs decreased in spleens of recipient mice after colonic inflammation was controlled. The results strongly suggested the safety of MDSCs transfer because the number of MDSCs in spleens of recipient mice did not increased in the end. In addition, as mentioned earlier, atorvastatin-derived MDSCs were mostly G-MDSCs and significantly inhibited the proliferation of T cells (74). Therefore, G-MDSCs were isolated from DSS-induced or T cell transfer-induced colitis mice after atorvastatin treatment and were intravenously injected to the corresponding colitis models. Inflammation was largely attenuated due to the suppressive function of atorvastatin-derived G-MDSCs, along with a significant decrease of CD4^+^ T cells. In 2016, Lee *et al.* examined the therapeutic effects of TGF-β-mediated, *in vitro* differentiated MDSCs (MDSCs^TGF-β^) on DSS-induced colitis (61). According to the study, colitis was alleviated after the intravenous injection of bone marrow-derived M-MDSCs or M-MDSCs^TGF-β^. However, transfer of M-MDSCs^TGF-β^ increased splenic Treg cells in colitis mice and were more efficient at repairing intestinal damages than the transfer of M-MDSCs. Consistently, M-MDSCs^TGF-β^ showed more localization in intestines, accounting for the stronger therapeutic effects. Ma *et al.* intravenously injected MDSCs isolated from wild type spleens to Arg^myeKO^ mice (the deficiency of Arg-1 in myeloid cells) with DSS-induced acute colitis and eventually prolonged the survival of these mice (64). Colitis in Arg^myeKO^ mice were more severe than in wild type mice due to the deficiency of Arg-1 in myeloid cells. After the injection of MDSCs, levels of IL17A instead of IL17F increased in Th17 cells, suggesting that MDSCs released Arg-1 to regulate Th17 cells to inhibit colitis progression in Arg^myeKO^ mice. Besides, as mentioned earlier, Chen *et al.* demonstrated that the injection of BQ123, an endothelin-A receptor antagonist, activated G-MDSCs in mice (72). Therefore BQ123-induced G-MDSCs were intravenously transferred to DSS-induced colitis and significantly alleviated inflammation. However, BQ123-induced G-MDSCs failed to alleviate colitis in recombination-activating gene 2 (RAG2) deficient mice with impaired T- and B-cell development, suggesting that BQ123-induced G-MDSCs relied on inhibiting T cells to alleviate inflammation. In addition to MDSCs transfer, transfer of extracellular vesicles, namely exosomes, which were secreted by MDSCs was also examined. Wang *et al.* isolated exosomes secreted by G-MDSCs from spleens of tumor-bearing mice and intraperitoneally injected G-MDSCs derived exosomes into DSS-induced acute colitis models (62). Due to the activity of Arg-1 in G-MDSCs derived exosomes, the frequency of Treg cells increased and the frequency of Th1 cells decreased respectively in mesenteric lymph nodes of the colitis mice. Therefore, the inflammation was significantly attenuated.

Although most studied confirmed the beneficial effects of MDSCs transfer, Kontaki *et al.* made contrast conclusions. The study first intrasplenically injected bone marrow derived MDSCs generated *in vitro* along with DO11.10 T cells or along with ovalbuminpulsed splenic dendritic cells into healthy mice (49). The proliferation and activation of T cells or dendritic cells were inhibited as expected *in vivo*. However, the suppressive effects of MDSCs either generated *in vitro* or isolated from TNBS-induced mice failed in TNBS-induced acute colitis mice after intravenous injection. The inflammation in acute colitis was augmented, at least not alleviated by transferred MDSCs. The discrepancy from the study conducted by Guan *et al.* (54) may be attributed to the differences between murine models. Guan *et al.* set up a more chronic colitis model by administrating TNBS twice over 1-week interval. Whereas in this study, mice were sacrificed after 3 to 5 days after a single transrectal injection of TNBS, establishing an acute colitis model. Therefore, the pro-inflammatory responses of T cells may not be fully activated yet so that suppressive effects of MDSCs were not observed in the acute colitis model. The study implied that MDSCs transfer may play a better therapeutic role as a maintenance therapy by preventing reactivation of colitis in remission.

## Conclusion and perspective

**Figure 1:**
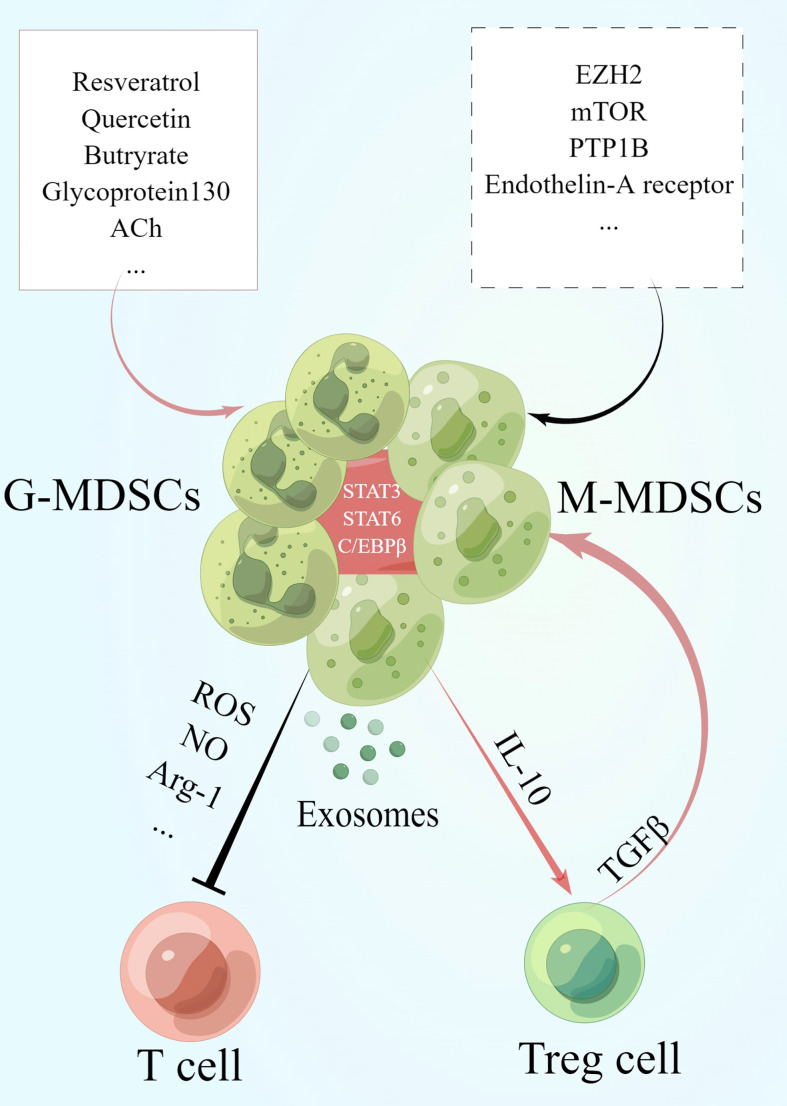
MDSCs and their immune regulatory effects on T cells. During the recovery phase of IBD, MDSCs exert their suppressive effects on T cells by secreting cytokines such as Arg-1, NO and ROS as well as exosomes expressing these cytokines. Meanwhile, MDSCs induce the increase of Treg cells *via* IL-10. Treg cells secret TGF-β to enhance the suppressive function of MDSCs in turn and specifically increase the number of M-MDSCs. The activation of signals in MDSCs such as STAT3, STAT6 and C/EBPβ plays a key role in its functioning. Besides, studies have revealed that the administration of resveratrol, quercetin, acetylcholine (ACh) and so on promotes the generation of MDSCs while the activation of zeste homolog 2 (EZH2), mTOR, protein tyrosine phosphatase 1b (PTP1B) and so on inhibits MDSCs. This graph is drawn by Figdraw.

Although the number of MDSCs increases in IBD, the suppressive function is not sufficient enough to inhibit colonic inflammation *in vivo*. Moreover, MDSCs can switch to be pro-inflammatory by differentiating to dendritic cells under acute inflammatory conditions. The dual role of MDSCs has also been reported in other inflammatory and autoimmune diseases like multiple sclerosis or rheumatoid arthritis (78–81). Besides, the immune regulatory effects of MDSCs on T cells can be influenced by various mediators *in vivo* (**Figure 1**). Based on the thesis, MDSCs-targeted therapies have been investigated and reported to alleviate IBD in most studies, including administration of bioactive molecules regulating MDSCs, and adoptive transfer of MDSCs or MDSCs-derived exosomes. Moreover, transfer of MDSCs has been shown to be more suitable as a maintenance therapy during the remission stage of colitis according to previous studies.

Challenges still exist in the field of MDSCs in IBD. First, given the similarity of MDSCs to monocytes and neutrophils both in phenotype and morphology, lack of specific markers remains one of the biggest controversies in the field of MDSCs. In fact, the definition of MDSCs is not consistent in current studies including M-MDSCs and G-MDSCs. Therefore, contrast conclusions have been made and it is difficult to conclude definitely that the disparity is due to different subsets of MDSCs or phases of the disease. Secondly, the function of M-MDSCs and G-MDSCs in IBD should be described respectively due to the heterogeneity observed in current studies and more studies *in vivo* are in need. Finally, although the safety and therapeutic effects of MDSCs transfer in experimental IBD have been demonstrated in several studies, it remains to be clear that how these transferred MDSCs influence inflammatory signals in local immune environment of colons and systemic immune system. Besides, the timing to administrate MDSCs transfer is crucial for the suppressive function of MDSCs considering the disease stage. A comprehensive understanding of the interaction between MDSCs and other immune mediators contributing to the intestinal inflammation should provide insights into the design of immunotherapeutic interventions.

## Author contributions

FZ and WG drafted the manuscript and designed the figure and Table. JS, ZS, and DC revised the manuscript. ZS and DC conceived the topic. All authors contributed to the article and approved the submitted version.

## Funding

This work was supported by the National Natural Science Foundation of China (Grant: 81871709, 82170533).

## Conflict of interest

The authors declare that the research was conducted in the absence of any commercial or financial relationships that could be construed as a potential conflict of interest.

## Publisher’s note

All claims expressed in this article are solely those of the authors and do not necessarily represent those of their affiliated organizations, or those of the publisher, the editors and the reviewers. Any product that may be evaluated in this article, or claim that may be made by its manufacturer, is not guaranteed or endorsed by the publisher.
